# Cesarean Scar – Unusual Site of Ectopic Pregnancy: A Case Report

**DOI:** 10.7759/cureus.5970

**Published:** 2019-10-22

**Authors:** Sumaya Al Gadeeb, Mohammed Al Gadeeb, Jumana Al Matrouk, Zainab Faisal, Afnan Mohamed

**Affiliations:** 1 Obstetrics and Gynecology, Maternity and Children Hospital, Al-Ahsa, SAU; 2 Radiology, King Fahd Hospital of the University, Al-Khobar, SAU

**Keywords:** cesarean scar, ectopic pregnancy

## Abstract

Ectopic pregnancy is a leading cause of maternal mortality in the first trimester. It may occur in different anatomic locations with fallopian tube being the most frequent. Cesarean-scar ectopic pregnancy is one of the rarest ectopic pregnancies. We report the case of a 44-year-old woman, gravida 5 para 4, who attended the antenatal clinic after her pregnancy was confirmed by positive urine testing. She underwent transvaginal ultrasound examination which identified the gestational sac with fetal pole and cardiac activity located in the anterior part of the lower uterine segment with empty uterine cavity. Magnetic resonance imaging (MRI) scan had confirmed the diagnosis of cesarean scar ectopic pregnancy. After through discussion on the management options, the patient was treated with intra-gestational sac injection of methotrexate. Four days after the procedure, she developed profuse vaginal bleeding and her hemoglobin showed a drop of 4.9 g/dL. She underwent emergency laparotomy with excision of the ectopic pregnancy. The patient tolerated the procedure well without complications. The serum β-human chorionic gonadotropin level was undetectable on the 35^th^ day after the methotrexate injection. Caesarean scar pregnancy is an unusual form of ectopic pregnancy. However, clinicians should have a high index of suspicion for this condition as it may result in serious complications, unless promptly managed. MRI is recommended particularly when transvaginal ultrasound scan is inconclusive.

## Introduction

Ectopic pregnancy refers to pregnancy in which the developing blastocyst implants at site other than the endometrial cavity. This type of pregnancy is the leading cause of pregnancy-related mortality in the first trimester as it is prone to life-threatening complications such as uterine rupture and hemorrhagic shock [1]. The most common site of ectopic pregnancy is the fallopian tube which accounts for 96% of all ectopic pregnancies [2]. However, an ectopic pregnancy may occur in different anatomic sites including the cervix, ovary, abdomen, myometrium, and previous cesarean scar. Cesarean-scar ectopic pregnancy is one of the rarest ectopic pregnancies occurring in approximately one in 2000 of pregnancies [3].

The diagnosis of ectopic pregnancy is based mainly on the measurement of serum β-human chorionic gonadotropin (β-hCG). Magnetic resonance imaging (MRI) might be used as an adjunct to ultrasound evaluation. Herein, we describe the case of 44-year-old woman who had a cesarean scar ectopic pregnancy that was confirmed by MRI scan. The patient was managed with medical treatment initially. However, she developed profuse bleeding because of ruptured ectopic pregnancy that required a surgical intervention.

## Case presentation

We present the case of a 44-year-old woman who attended the antenatal care clinic after six weeks of amenorrhea. The pregnancy was confirmed by positive urine β-hCG testing. She did not have any vaginal bleeding. She had four live births by low-transverse cesarean section with no previous miscarriages or ectopic pregnancies. The first two cesarean section deliveries were due to failure to progress. Her last pregnancy was four years ago. The past medical history revealed ulcerative colitis managed by mesalamine.

**Figure 1:**
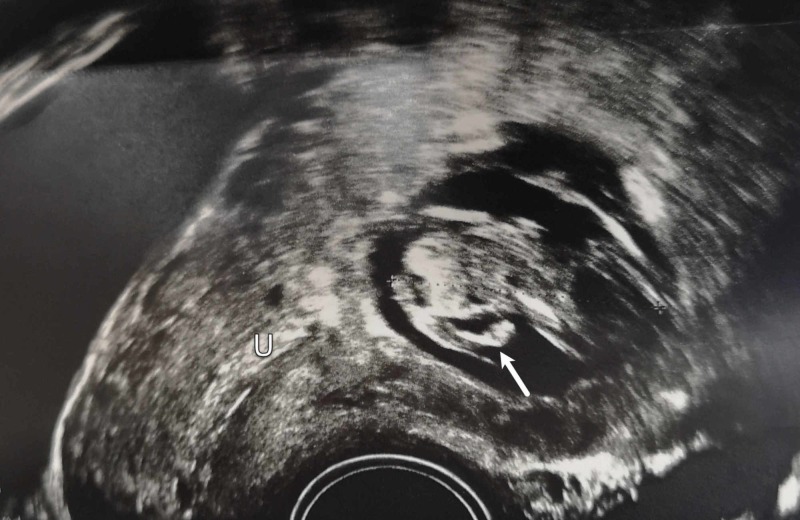
Transvaginal Ultrasound Scan Transvaginal ultrasound image showing the presence of gestational sac with fetal pole (arrow) located in the anterior part of the lower uterine segment with empty uterine cavity (U).

**Figure 2:**
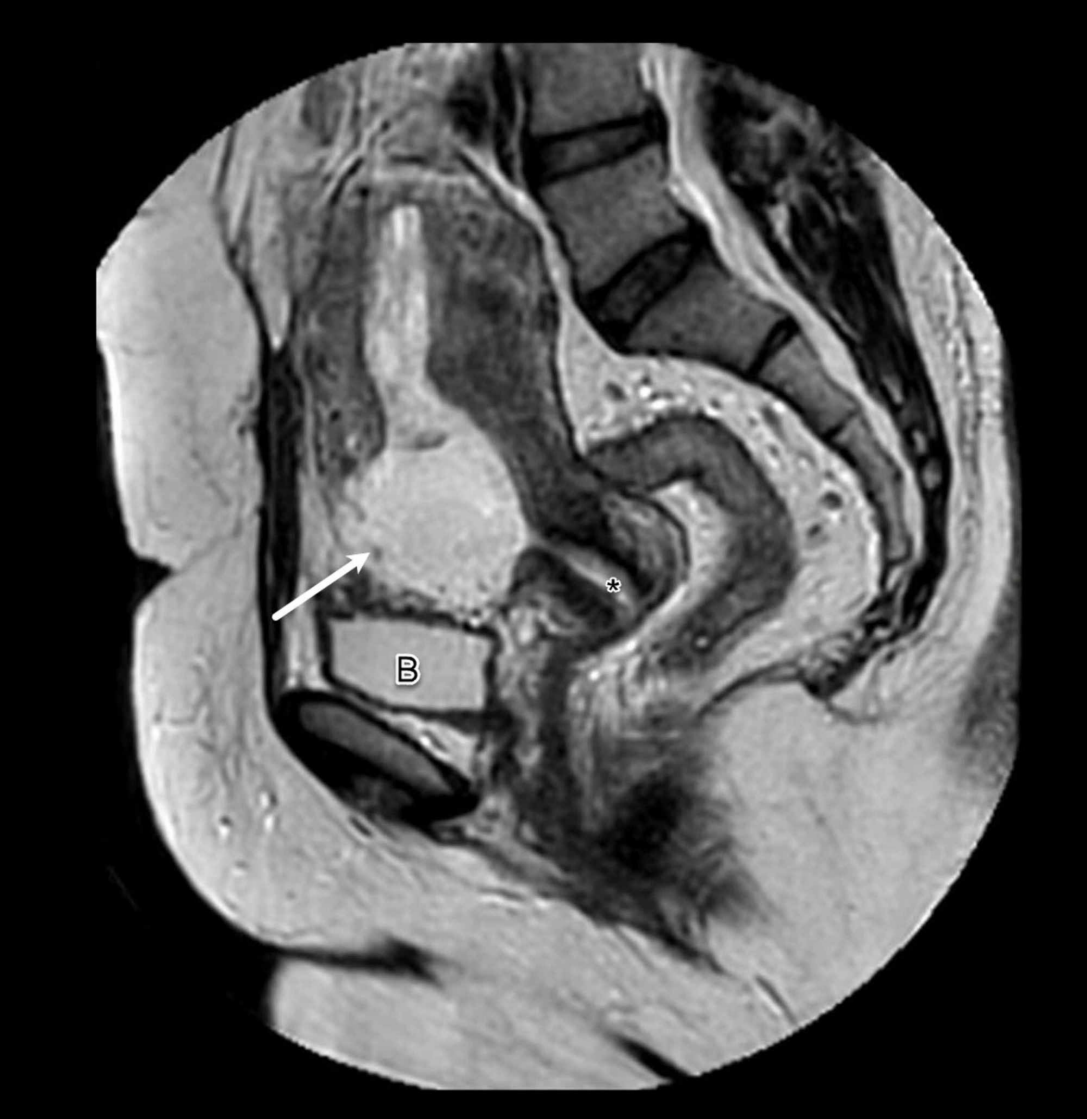
MRI Scan of Cesarean Ectopic Pregnancy Sagittal T2-weighted MRI of the pelvis demonstrating a gestational sac (arrow) implanted within the anterior wall of the lower uterine segment in the region of previous cesarean scar. The uterine cavity and cervical canal were empty (asterisk). The anterior wall anterior to the gestational sac is thinned out while the posterior wall is seen communicating with the endometrial cavity. Bladder wall integrity was preserved (B).

On examination, she had a normal blood pressure of 125/76 mmHg, a pulse rate of 89 beats/min and body temperature of 37.1 °C. Her abdomen was soft, lax and non-tender. Her cardiorespiratory and neurological systems were normal. The results of pelvic examination were normal. Laboratory investigations revealed hemoglobin level of 12.7 g/dL and β-hCG level of 93,788 mIU/mL. The patient underwent transvaginal ultrasound examination which identified the gestational sac with fetal pole and cardiac activity located in the anterior part of the lower uterine segment near the cervicouterine junction along with empty uterine cavity (Figure 1). The crown-rump length of the embryo was 2.10 cm, consistent with gestational age of eight weeks. On Doppler examination, hyperechoic rim of choriodecidual reaction with prominent vascularity was noted. As the findings were suggestive of cesarean section scar ectopic pregnancy, the patient underwent MRI scan which confirmed the diagnosis of ectopic scar pregnancy (Figure 2). The MRI findings demonstrated a gestational sac measuring 3.5 × 2.9 × 3.5 cm implanted in the anterior wall of the lower uterine segment in the region of the scar of previous cesarean section. The gestational sac had T1-isointense and T2-hypointense fetal pole and was surrounded by decidual reaction. Posteriorly, the gestational sac was communicating with the endometrial cavity.

The patient was counselled regarding management options of ectopic pregnancy including medical and surgical treatment with thorough explanations of advantages and disadvantages of each choice. She chose medical treatment because of her strong desire to preserve her uterus and maintain future fertility. Therefore, she received an 80 mg of methotrexate into the ectopic gestational sac under transvaginal ultrasound-guided technique. There was no internal bleeding or lower abdominal pain noticed following the procedure. The patient was admitted to the ward for observation. A repeat serum quantitative β-hCG after two days from the injection revealed a level of 50,057 mIU/ml, showing 46% drop from the initial result. Four days following the procedure, the patient complained of lower abdominal pain and profuse vaginal bleeding. On physical examination, lower abdominal tenderness without rigidity or guarding was noted. Laboratory investigations revealed a 4.9 g/dL drop in her hemoglobin level.

**Figure 3:**
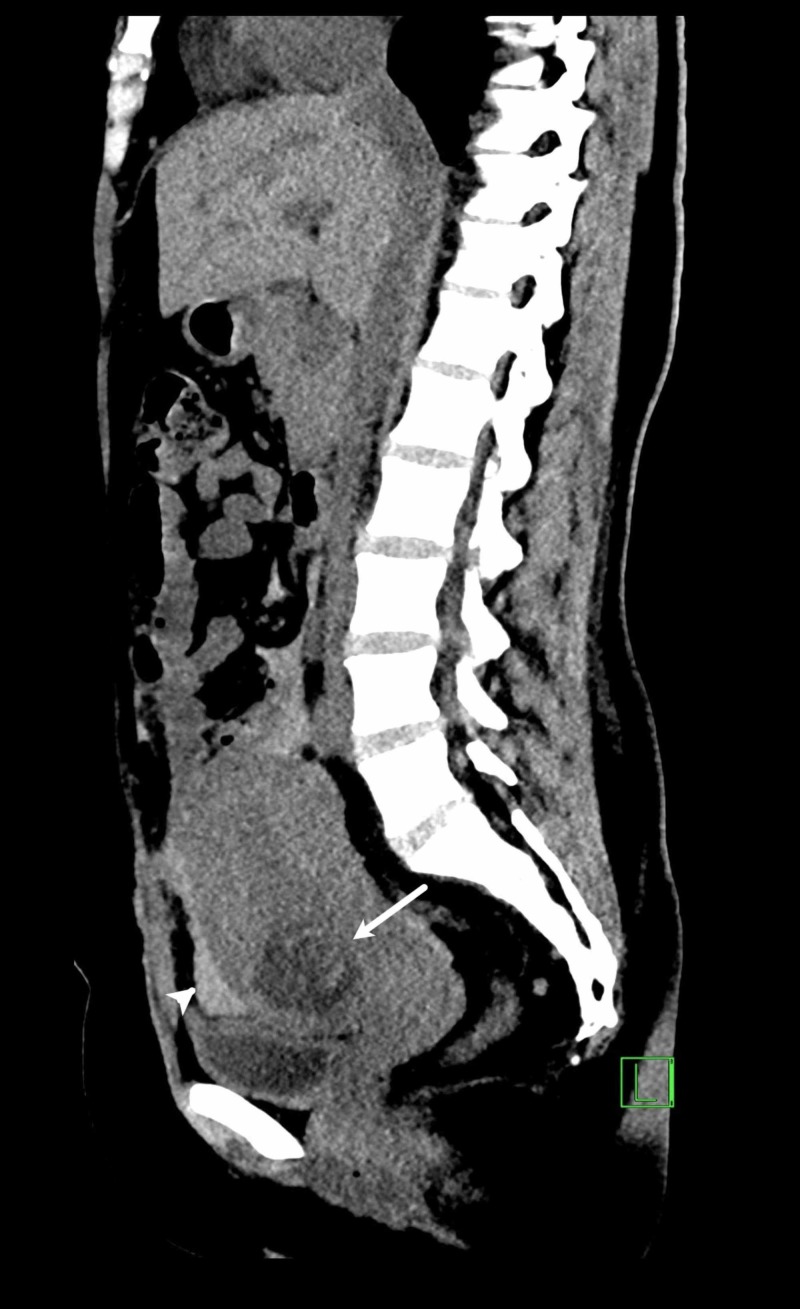
CT Scan for Hemoperitoneum Sagittal CT scan image demonstrating the gestational sac (arrow) and high-density fluid collection (arrow head) suggestive of hemoperitoneum.

Urgent abdominal computed tomography scan was performed and it demonstrated a bulky uterus with a cystic structure that was surrounded by hyperdense attenuation at the junction of middle and lower thirds of the uterus along with the presence of high volume of high-density fluid in the abdominopelvic cavity (Figure 3). These findings indicated hemoperitoneum due to a ruptured ectopic scar pregnancy. Therefore, the patient was resuscitated and prepared for emergency laparotomy for excision of the ectopic scar pregnancy with possible hysterectomy. During surgical exploration, a soft mass was seen at the site of previous cesarean scar. An incision was made over the mass and the products of conception were removed. The bulge was noted to be communicating with the uterine cavity. The edges of the scar tissue were excised and freshened. The obtained tissue was sent for histopathological examination which revealed the presence of products of conception with phenotypically male fetus of seven weeks of age. The patient tolerated the procedure well and had uneventful recovery. The serum β-hCG was 10,894 mIU/ml on the first post-operative day. She was discharged on the fifth post-operative day in a stable condition. The patient had weekly clinical evaluation and measurement of serum β-hCG level. She was asymptomatic and had no active complaints. Her serum β-hCG was undetectable on the 35th day after the methotrexate injection.

## Discussion

The presented case demonstrated a cesarean scar ectopic pregnancy which was managed with local methotrexate injection and developed a potentially life-threatening hemorrhage. Cesarean scar ectopic pregnancy is one of the long-term complications of cesarean deliveries. The first case of scar ectopic pregnancy was reported by Larsen and Solomon in 1978 [4]. Although considered a rare type of pregnancy, the incidence of cesarean scar ectopic pregnancy is increasing owing to the increasing rates of cesarean deliveries worldwide [5, 6].

There are two types of cesarean scar ectopic pregnancy. In the first type, the implanted gestational sac grows towards the cervicoisthmic space or the uterine cavity. Such pregnancy might proceed to term with a viable fetus but it has an increased risk of life-threatening massive postpartum hemorrhage from the implantation site [7]. In the second type, the deeply implanted gestational sac grows towards the serosal surface of the uterine wall. This type carries the risk of rupture and hemorrhage during the first trimester of pregnancy.

The exact pathogenesis of cesarean scar ectopic pregnancy is unclear. It has been postulated that the blastocyst invades into the myometrium through a microscopic uterine dehiscent tract which is related to a previous uterine surgery (e.g., cesarean section). However, this hypothesis does not explain the occurrence of scar ectopic pregnancy in the absence of previous uterine surgeries [8]. For such cases, it is suggested that scar pregnancy may occur due to a trauma occurred during manual extraction of placenta or during assisted reproduction techniques [9, 10]. In our case, the patient had four previous cesarean section deliveries which predisposed her for ectopic pregnancy. Interestingly, the risk of cesarean scar pregnancy appears to be unrelated to the number of previous cesarean deliveries [5, 6].

The clinical presentation of a cesarean scar ectopic pregnancy ranges from vaginal bleeding to uterine rupture and hypovolemic shock [11]. Hence, the early and accurate diagnosis of scar pregnancy is crucial. The diagnosis is typically made based on the ultrasound evaluation of the uterus. The proposed diagnostic criteria of scar pregnancy include the following [12, 13]:

1. Presence of gestational sac in the anterior part of the lower uterine segment.

2. An empty uterus and cervical canal.

3. Absence of myometrium between the bladder wall and the gestational sac. This is essential to differentiate scar pregnancy from cervical pregnancy.

The MRI can be used as an adjunct to ultrasound scanning, as it helps to confirm the diagnosis when the ultrasound findings were inconclusive [14, 15]. Because of improved differentiation of soft tissue structures and spatial resolution, MRI clearly shows the gestational sac in the anterior lower uterine segment and it can assess the possibility of myometrial invasion and bladder involvement. It is also used to measure the gestational sac volume and evaluate the pelvic anatomy which can improve the intra-operative orientation.

Given the rarity of scar pregnancies, much of the information regarding the management has been derived from small observational studies and case reports. The optimal treatment of cesarean scar pregnancy remains to be undefined. However, the treatment plan should be tailored to the individual patient considering the patient’s preference and desire for future fertility, the size of the gestational sac, the estimated gestational sac, and the hemodynamic condition of the patient. The management options include wedge resection of the ectopic pregnancy via laparotomy or laparoscopy, hysteroscopic excision, local injection of potassium chloride, and local or systemic methotrexate administration [16]. In our case, the patient opted for the medical treatment initially. However, her course was complicated by rupture and hemorrhage and she required emergency laparotomy for excision of the ectopic pregnancy.

The risk of recurrent cesarean scar ectopic pregnancy is low [17]. However, even with subsequent intrauterine pregnancy, the patient is at risk of adherent placenta, uterine rupture, and fetal or maternal death.

## Conclusions

Caesarean scar pregnancy is an unusual form of ectopic pregnancy. However, clinicians should have a high index of suspicion for this condition as it may result in serious complications, unless promptly managed. MRI is recommended particularly when transvaginal ultrasound scan is inconclusive.
